# Long-term impact of scheduled regular endoscopic interventions for patients with primary sclerosing cholangitis

**DOI:** 10.1097/HC9.0000000000000494

**Published:** 2024-09-03

**Authors:** Burcin Özdirik, Wilfried Veltzke-Schlieker, Jule Marie Nicklaus, Hilmar Berger, Daniel Schmidt, Silke Leonhardt, Volker Penndorf, Andreas Adler, Tobias Müller, Alexander Wree, Frank Tacke, Michael Sigal

**Affiliations:** 1 Department of Hepatology and Gastroenterology, Campus Virchow Klinikum (CVK) and Campus Charité Mitte (CCM), Charité Universitätsmedizin Berlin, Berlin, Germany; 2 Berlin Institute of Health at Charité – Universitätsmedizin Berlin, BIH Biomedical Innovation Academy, BIH Charité Clinician Scientist Program, Charitéplatz 1, Berlin, Germany; 3 Berlin Institute for Medical Systems Biology, Max Delbrück Center for Molecular Medicine, Berlin, Germany

## Abstract

**Background:**

Primary sclerosing cholangitis (PSC) is associated with biliary obstructions that can require endoscopic retrograde cholangiopancreatography (ERCP). While the beneficial effects of ERCP are well documented, follow-up interventional strategies are less defined, and their long-term impact is debated.

**Methods:**

We evaluated the outcome of a scheduled program of ERCP-guided interventions that have been developed and implemented at our tertiary liver center for more than 20 years. Within our center, follow-up ERCPs were performed at regular intervals to treat previously detected morphological stenosis independent of clinical symptoms. We calculated the transplant-free survival (TFS) of patients who were enrolled in the scheduled ERCP program and compared it to patients who received follow-up ERCPs only on clinical demand. Moreover, we documented the occurrence of hepatic decompensation, recurrent cholangitis episodes, hepatobiliary malignancies, and endoscopy-related adverse events.

**Results:**

In our retrospective study, we included 201 patients with PSC who all received an ERCP. In all, 133 patients received scheduled follow-up ERCPs and 68 received follow-up ERCPs only on demand. The rates of TFS since initial diagnosis (median TFS: 17 vs. 27 y; *P* = 0.020) and initial presentation (median TFS: 16 vs. 11 y; *P* = 0.002) were higher in patients receiving scheduled versus on-demand ERCP. Subgroup analysis revealed that progression in cholangiographic findings between the first and second ERCP was associated with a poorer outcome compared to patients without progression (17 y vs. undefined; *P* = 0.021).

**Conclusion:**

In conclusion, we report the outcome data of a scheduled follow-up ERCP program for patients with PSC in an experienced high-volume endoscopy center. Our data suggest the initiation of multicenter randomized controlled prospective trials to explore the full potential of regular endoscopic follow-up treatment as a strategy to prevent disease progression in patients with PSC.

## INTRODUCTION

Primary sclerosing cholangitis (PSC) is an immune-related cholangiopathy marked by biliary inflammation, cholestasis, and multifocal bile duct strictures. PSC is associated with high rates of progression to end-stage liver disease as well as a substantial risk of cholangiocarcinoma (CCA), gallbladder cancer, and colorectal carcinoma. Currently, liver transplantation (LT) is the only curative treatment option.^1,2^ Patients with PSC have multiple biliary strictures in the intrahepatic and extrahepatic bile ducts. Extrahepatic strictures are more frequently associated with episodes of jaundice, bacterial cholangitis, cholelithiasis, and neoplastic development.^3,4^ Endoscopic retrograde cholangiography-pancreatography (ERCP) has become the primary management strategy for these complications in patients with PSC due to its diagnostic accuracy as well as its sampling and therapeutic options.^3,5^ According to current European guidelines, endoscopic treatment is recommended for patients with “relevant strictures,” which are high-grade strictures (> 75% reduction of duct diameter) in the common bile duct or hepatic duct on magnetic resonance cholangiopancreatography (MRCP)-imaging with symptoms or signs of acute cholangitis or obstructive cholestasis.^6^ The new-onset or progression of symptoms such as pruritus, unexplained weight loss, rise in cholestatic enzyme levels, recurrent cholangitis episodes, as well as new onset of bile strictures and progression of bile duct strictures should lead to diagnostic endoscopic treatment.^6,7^ While short-term improvements of all these parameters upon ERCP are well established, there are only limited data on the long-term impact of endoscopy.^8–11^ More specifically, the need for follow-up procedures and their exact implementation—that is, follow-up procedures only on demand upon re-occurrence of symptoms versus scheduled procedures at predefined intervals independently of clinical symptoms—is not well studied, although a recent retrospective single-center report suggests benefits of a scheduled ERCP program.^12^

Here, we report the long-term outcome data of the scheduled endoscopic surveillance and management program, which has been developed and implemented over the last 20 years at our tertiary liver transplantation center.

## METHODS

### Study design and study population

**FIGURE 1:**
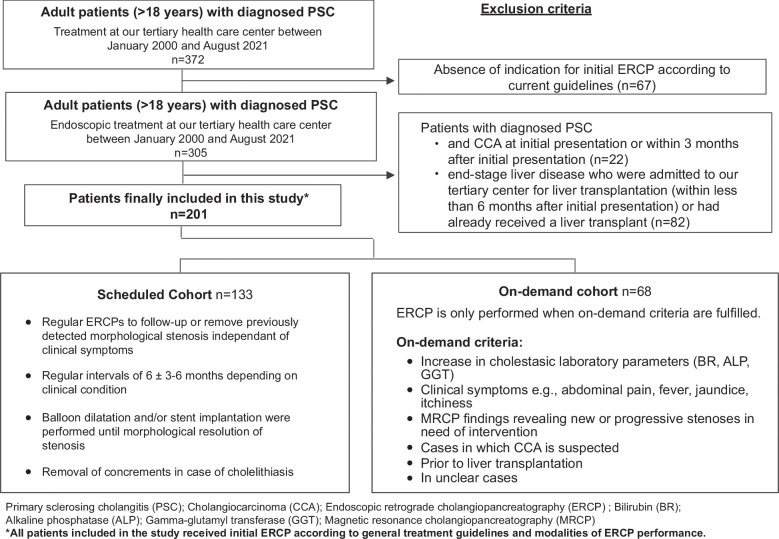
Overview of patients included in the study and characteristics of the scheduled versus on-demand cohort. Flowchart with an overview of all patients included in this study, the exclusion criteria, and the characteristics of the scheduled and on-demand cohort. Abbreviations: ALP, alkaline phosphatase; BR, bilirubin; CCA, cholangiocarcinoma; ERCP, endoscopic retrograde cholangiopancreatography; GGT, gamma-glutamyl transferase; MRCP, magnetic resonance cholangiopancreatography; PSC, primary sclerosing cholangitis.

In our retrospective analysis, we explored the outcome of our scheduled endoscopic surveillance and management program over more than 20 years. Three hundred and seventy-two adult patients (> 18 y) with diagnosed PSC were treated at our tertiary liver center between January 2000 and August 2021. Of these, 305 patients (> 18 y) with diagnosed PSC received endoscopic treatment and were initially assessed as eligible for this study. After the exclusion of 104 patients in total, our final study cohort comprised 201 patients. Twenty-two patients had to be excluded due to the presence of CCA at initial presentation or within 3 months after initial presentation and 82 patients were excluded due to the presence of end-stage liver disease with the necessity of LT within 6 months after initial presentation or because they had already received a liver transplant (Figure 1). Patients who were assigned to our program cohort, underwent elective ERCP procedures at regular intervals to follow-up previously detected morphological stenosis independent of clinical symptoms. Patients who were not able to be part of our scheduled program (in case of refusal to participate, nonadherence to the scheduled program, for example. due to geographical reasons) were retrospectively assigned to the on-demand cohort and received endoscopic interventions only when fulfilling the on-demand criteria. ERCP reports of all patients were analyzed and evaluated. Medical data were extracted from electronic medical charts. Diagnosis of PSC was confirmed in accordance with current guidelines using a combination of clinical, biochemical, and cholangiographic (MRCP and/or ERCP) features.^13,14^ Acute cholangitis was defined based on a combination of systemic inflammation, cholestasis, and imaging.^15^ Only cholangitis episodes requiring hospitalization and i.v. antibiotic treatment were included in our analysis. Hepatic decompensation was defined as at least 1 episode of ascites, spontaneous bacterial peritonitis (SBP), hepatic encephalopathy (HE), variceal bleeding, and/or hepatorenal syndrome.^16^ Diagnosis of hepatobiliary malignancies was confirmed histologically. The definition criteria for the procedure-associated adverse events we used are listed in Supplemental Table 1, http://links.lww.com/HC9/B11.^17^ The Model for End-Stage Liver Disease (MELD) score and Mayo risk score were calculated according to published algorithms.^18,19^ LT allocation in Germany is regulated within the Eurotransplant system, which was changed in 2006 from a waiting time–based allocation system to an urgency-based system using the MELD score.^20^ Time of initial presentation was defined as the time span from the first presentation at our tertiary liver center to the present, and the time of initial diagnosis was defined as the duration from the initial diagnosis to the present. The time of the initial ERCP was defined as the time span from the first ERCP to the present. The time of the first ERCP and time of initial presentation were not always identical dates since many patients underwent surveillance at our outpatient clinic until indication criteria for an initial ERCP were fulfilled. The minimum follow-up length time was defined as at least 5 months after the initial presentation.

### Scheduled ERCP program

We have retrospectively analyzed the outcome data of patients who were included in our ERCP program as well as of patients who received ERCP only on demand. Patients who were assigned to our program cohort underwent elective ERCP procedures at regular intervals to follow-up on previously detected morphological stenosis independent of clinical symptoms. Initial ERCP was performed in accordance with guideline recommendations and general treatment modalities, which include (i) the development of clinically relevant or worsening symptoms such as pruritus, jaundice, and cholangitis; (ii) a fast rise in cholestatic enzyme levels; or (iii) the new-onset or progression of biliary strictures in MRCP-imaging.^5,14^ Follow-up ERCPs were performed at regular intervals of 6 months. Intervals were shortened to 3 months in case of cholangiographic, clinical (itching or other symptoms) and/or biochemical progression. Cholangiographic progression was defined as an increase in the number of stenoses, progression in the extent of stenosis, the increase in rarefication of the bile duct system, and the necessity of a stent implantation. In cases with recurrent response to treatment or stable cholangiographic, clinical, and biochemical findings, intervals were prolonged to 9-12 months. A relevant stenosis was defined as high-grade stricture on imaging in the common bile duct or hepatic ducts according to current european guidelines.^6^ Balloon dilatation until morphological resolution of stenosis was the standard procedure, while in some cases, stent implantation was performed. Moreover, concrements in cases of cholelithiasis were removed (Figure 1). Of the 133 patients in the planned cohort, all but 18 adhered to our planned intervals. The remaining 18 patients extended the interval beyond the planned procedure but then returned to regular endoscopic surveillance.

Patients who were not able to be part of our scheduled program (in case of refusal to participate, nonadherence to the scheduled program, for example, due to geographical reasons) were retrospectively assigned to the on-demand cohort and received endoscopic interventions only when fulfilling the on-demand criteria. Again, initial ERCP was performed in accordance with guideline recommendations and general treatment modalities, which include (i) the development of clinically relevant or worsening symptoms such as pruritus, jaundice, and cholangitis; (ii) a fast rise in cholestatic enzyme levels; or (iii) the new-onset or progression of biliary strictures in MRCP-imaging.^5,13^ Further, initial ERCP was performed for diagnostic purposes, such as suspicion of CCA, before liver transplantation and in unclear cases^14,21,22^ (Figure 1).

Both patient cohorts were offered the same guideline-compliant surveillance alternatives including regular laboratory testing and surveillance through imaging (ultrasound and/or MRCP) independent from performed endoscopic interventions. In case patients were not treated at our tertiary liver center on a regular basis, for example, due to geographical reasons, they were offered the same surveillance program at other local sites in a closer distance to their homes with a direct connection to our tertiary liver center. Since recent studies confirmed that early tumor detection in patients with PSC is not associated with endoscopic interventions, MRCP-imaging is an essential part of our current PSC surveillance program.^23^

### Endoscopic procedures and preinterventional and postinterventional surveillance

All patients with PSC undergoing ERCP received peri-interventional antibiotics. The standard regimen in our institution was the i.v. administration of a single dose (2 g) of ceftriaxone 30–60 minutes before each procedure. If needed, adaption according to previous bile culture results was performed, and in case of known allergies, ciprofloxacin or ampicillin/sulbactam were used. Propofol, midazolam and piritramide were used for sedation. At entry, the biliary system was visualized. Standardized cannulation of the common bile duct and endoscopic sphincterotomy at initial ERCP were performed. In patients with relevant stenosis, we applied the dilatation method with stepwise balloon dilatations of the strictures starting from 4 mm. To limit the risk of perforation in the biliary tract, our advanced endoscopists performed balloon dilatations up to a maximum of 6 mm in the common bile duct and 4 mm in the hepatic ducts. In the case of stent implantation, a plastic stent (10F) was placed for 2–4 weeks. Additional stenting was performed only rarely after 2016, when the evidence against stenting in PSC was communicated.^24^ The morphological resolution was determined by either no remaining identifiable sign of the previous stricture on cholangiography or a sole minor narrowing of the bile duct in the form of a residuum of the previous stricture. However, all cases captured an unhindered passage of biliary catheters and an unobstructed passage of contrast medium through the balloon-dilated biliary segment. Following the endoscopic intervention, patients were admitted to the ward to be monitored for at least 24 hours.

### Clinical study endpoints

The primary end point of the study was transplant-free survival (TFS) in the scheduled versus on-demand cohort. TFS was defined as survival free of liver-related death or LT. The secondary endpoints were defined as the occurrence of:

- episodes of hepatic decompensation (defined as at least one of the following features: ascites, SBP, hepatic encephalopathy variceal bleeding, or hepatorenal syndrome) since initial presentation
- (recurrent) acute cholangitis episodes with the necessity of hospitalization and i.v. antibiotic treatment
- hepatobiliary malignancies (CCA and HCC)
- procedure-associated adverse events: post-ERCP–cholangitis/post-ERCP-pancreatitis,post-ERCP–bleeding/post-ERCP–perforation (of the duodenum and/or extrahepatic bile duct).

### Consent

Our retrospective study protocol was reviewed and approved by the institutional ethics committee of the Charité University Medicine (ethical approval number EA1/142/21) and was carried out in accordance with the Declaration of Helsinki. All authors had access to the study data and reviewed and approved the final manuscript.

### Statistical Analysis

Frequencies were compared using the chi-square test. Nonparametric data were compared using the Mann-Whitney *U*-test. Kaplan-Meier curves were used to display the impact of a specific parameter on TFS and overall survival. A log-rank test was performed to assess whether significant differences exist between cohorts. Analysis of survival rates between cohorts at specific time points was performed using chi-square test. Cox proportional HR models were used to determine factors that independently affected the risk of reduced TFS. Alkaline phosphatase (ALP) was categorized as above or below 2.5 × of the upper limit of normal (ULN) according to the UK-PSC Risk Score.^25^ We included age at initial diagnosis and IBD due to their previously described prognostic values, however, it should be noted that the role of IBD for the progression of liver disease in PSC is still under debate. ^26–30^.We performed propensity score matching adjusting for covariates such as gender, age at initial diagnosis, presence of inflammatory bowel disease (IBD), presence of relevant stenosis at initial ERCP, MELD score, and ALP at the initial presentation in our patient cohort treated either in the scheduled program or on demand. Pairs of patients with similar propensity scores and differing cohort selection status were selected by R package MatchIt after patients with missing values in any of the selected covariates were excluded.^31^ All statistical analyses were performed with Prism (version 7.03; GraphPad, La Jolla, California, USA), SPSS 26 (SPSS, Chicago, IL, USA), and RStudio (v1.2.5033, RStudio, Inc., Boston, MA, USA). A *P*-value of < 0.05 was considered statistically significant (*P < 0.05, **P < 0.01, ***P < 0.001, ****P < 0.0001).

## RESULTS

### Baseline characteristics

Our final cohort comprised 201 patients; 133 patients belonged to the scheduled cohort and 68 patients received ERCP procedures only on demand. Baseline clinical and ERCP characteristics are shown in Table 1. Gender, age, time between initial diagnosis and initial presentation, presence of overlap syndrome or IBD, and treatment with ursodeoxycholic acid did not show significant differences between both cohorts. The average number of ERCP procedures was higher in the scheduled cohort compared to the on-demand cohort (median 7 [range 2–40] vs. 2 [1–12]; *P*<0.001). At initial ERCP, a relevant stenosis could be detected in more patients in the scheduled cohort (80% vs. 66%; *P* = 0.031). The morphology of stenosis (intrahepatic/extrahepatic manifestation) was equally distributed between both cohorts. In addition to balloon dilatation, stenting was performed in 18% of patients (17% vs. 21%, not significant) and 5% of interventions in total (6% vs. 9%, not significant).

**TABLE 1:** Baseline patient and disease characteristics of the patients with PSC

	All patients n = 201	Scheduled cohort n = 133	On-demand cohort n=68	*P*-value on-demand vs. scheduled cohort
Gender				
Male	141 (70)	92 (69)	49 (72)	0.672
Female	60 (30)	41 (31)	19 (28)	—
Median age at initial diagnosis (range)	31 (10, 67)	31 (10, 67)	31 (10, 65)	0.804
Median time between initial diagnosis and initial presentation (y)	3 (0, 36)	2 (0, 36)	4 (0, 23)	0.120
Follow-up (y)	8 (0, 21)	7 (0, 21)	10 (0, 21)	0.123
Mean BMI (SD)	24 (± 3.9)	24 (± 3.9)	23 (± 3.8)	0.021
Comorbidities				
Arterial hypertension	41 (20)	28 (21)	13 (19)	0.747
Diabetes	8 (4)	5 (4)	3 (4)	0.823
Asthma/COPD	8 (4)	4 (3)	4 (6)	0.324
Overlap syndrome with AIH	22 (11)	13 (10)	9 (13)	0.457
Presence of liver cirrhosis	95 (48)	56 (41)	40 (59)	**0.026**
Presence of IBD	154 (77)	97 (73)	57 (84)	0.084
Ulcerative colitis	129 (64)	78 (59)	51 (75)	—
Crohn’s disease	19 (10	15 (11)	4 (6)	—
Undetermined	6 (3)	4 (3)	2 (3)	—
UDCA^a^	196 (99)	130 (99)	66 (99)	0.627
Median number of ERCPs performed per patient (range)	5 (1, 40)	7 (2, 40)	2 (1, 12)	**<0.001**
Relevant stenosis^b^	150 (75)	106 (80)	44 (66)	**0.031**
Manifestation^c^				0.389
Exclusively intrahepatic	69 (35)	42 (32)	27 (41)	—
Exclusively extrahepatic	30 (15)	22 (17)	8 (12)	—
Intrahepatic and extrahepatic	100 (50)	69 (52)	31 (47)	—
Stent implantation per patient	36 (18)	22 (17)	14 (21)	0.479
Stent implantation per intervention	52/794 (7)	35/615 (6)	17/179 (9)	0.471
Median baseline laboratory parameters (Min-Max)				
** **Bilirubin [mg/dL]	0.8 (0.19, 27.7)	0.8 (0.19, 11.7)	0.85 (0.29, 27.7)	0.838
ALT [U/L]	59 (9, 599)	64 (9, 599)	53 (14, 346)	0.067
AST [U/L]	51 (17, 384)	54 (17, 384)	46 (19, 237)	0.167
ALP [U/L]	217 (49, 872)	218 (61, 798)	208 (49, 872)	0.917
GGT [U/L]	184 (8, 1463)	191 (10, 1463)	163 (8, 1312)	0.279
Creatinine [mg/dL]	0.83 (0.39, 1.7)	0.84 (0.4, 1.7)	0.8 (0.5, 1.23)	0.141
INR	1 (0.85, 2.55)	1 (0.9, 1.5)	1 (0.9, 2.55)	0.529
MELD	7 (6, 28)	7 (6, 20)	8 (6, 28)	0.078
MRS	−0.17 (−2.2, 2.78)	−0.26 (−2.2, 2.78)	0.16 (−2, 2.64)	0.412

*Notes:* Data are n (%) of patients, if not indicated otherwise. The percentages were rounded and may not sum 100%. Significant results (*P* < 0.05) are shown in bold type.

Laboratory reference values: Bilirubin < 1.2 mg/dL, ALT < 31 U/L, AST 35 U/L, ALP 35–105 U/L, GGT 5–36 U/L, creatinine 0.5–0.96 mg/dL, INR 0.9–1.25.

^a^ UDCA treatment was analyzed in 260 patients; 131 patients in the scheduled cohort and 67 patients in the on-demand cohort.

^b^ Relevant stenosis was analyzed in 200 patients; 133 patients in the scheduled cohort and 67 patients in the on-demand cohort.

^c^ Manifestation of stenosis was analyzed in 199 patients; 133 patients in the scheduled cohort and 66 patients in the on-demand cohort.

Abbreviations: AIH, autoimmune hepatitis; ALP, alkaline phosphatase; ALT, alanine transaminase; AST, aspartate aminotransferase; BMI, body mass index; COPD, chronic obstructive pulmonary disease; ERCP, Endoscopic retrograde cholangiopancreaticography; GGT, gamma-glutamyl transferase; IBD, inflammatory bowel disease; INR, international normalized ratio; MELD, Model for End-Stage Liver Disease; MRS, Mayo risk score; UDCA, ursodeoxycholic acid.

Cholestatic laboratory parameters, MELD score, and Mayo risk score at the initial presentation were similar in both cohorts. The median follow-up was 8 years (0–21), 7 (0–21) years in the scheduled cohort, and 10 (0–21) years in the on-demand cohort (*P* = 0.123) (Table 1). Further, lost-to follow-up patient numbers were similar in both cohorts (10 [8%] vs. 7 [(10%].

### Outcome analysis and TFS

**FIGURE 2:**
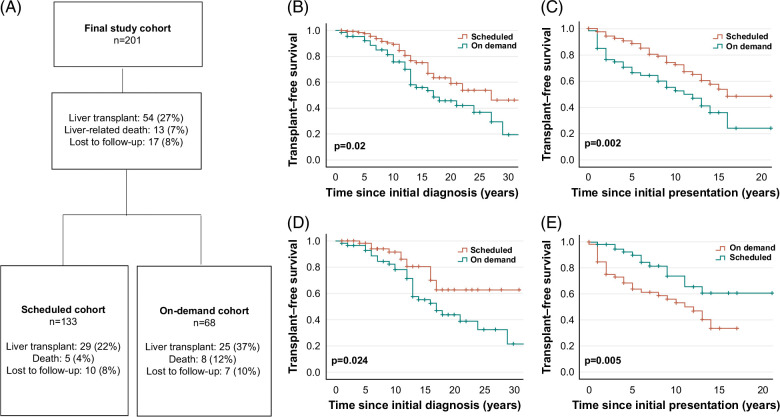
Outcome analysis and TFS in the scheduled versus on-demand cohort. Flowchart with an overview of our outcome analysis results in the scheduled versus on-demand cohort (A). Kaplan-Meier analysis of TFS in the scheduled (red curve) versus on-demand cohort (blue curve) since initial diagnosis revealed a beneficial outcome for the scheduled cohort (median TFS: 27 y vs. 17 y; *P* = 0.02) (B). Our analysis revealed a significantly longer TFS since the initial presentation for patients in the scheduled cohort (median TFS: 16 y vs. on demand: 11 y; *P* = 0.002). (C). To minimize selection bias, we performed propensity score matching and adjusted covariates in the scheduled and on-demand cohorts. Kaplan-Meier analysis of the matched cohorts revealed a superior TFS of patients in the scheduled cohort (red curve) compared to the on-demand cohort (blue curve) since initial diagnosis (median TFS undefined vs. 17 y; *P* = 0.024) (D) and since initial presentation (median TFS: undefined vs. 12 y; *P* = 0.005) (E); (*P < 0.05; **P < 0.01; ***P < 0.001). Abbreviations: TFS, transplant-free survival.

During the observation period, 27% of patients received a liver transplant and 7% died from a liver-related cause. Our results reveal that significantly more patients in the on-demand cohort underwent LT (22% vs. 37%; *P* = 0.024) and died due to a liver-related death (4% vs. 12%; *P* = 0.029) (Figure 2A).

The median TFS in our entire PSC study cohort was 22 years since initial diagnosis and 14 years since the initial presentation. The median TFS since initial diagnosis was 27 years in the scheduled cohort compared to 17 years in the on-demand cohort (*P* = 0.02). The 15-year survival rate was 75% versus 56%, respectively, *P* = 0.003 (Figure 2B). Further, our analysis revealed a significantly longer median TFS since initial presentation for patients in the scheduled cohort (16 y vs. 11 y; *P* = 0.002). The 5-year survival rate after initial presentation was 89% versus 66%, *P* < 0.001 (Figure 2C). In line, median TFS since first ERCP was significantly better in the scheduled cohort (16 y vs. 9 y; *P* < 0.001) (Supplemental Figure 1, http://links.lww.com/HC9/B11). To reduce bias due to confounding variables, we computed a propensity score based on gender, age at initial diagnosis, presence of IBD, presence of relevant stenosis at initial ERCP, baseline MELD score, and baseline ALP. After a comparison of its distribution in both cohorts, 120 patients (ie, 60 patients per group) could be matched. Notably, Kaplan-Meier analysis of the matched cohorts confirmed a beneficial outcome since initial diagnosis (undefined vs. 17 y; *P* = 0.024) and initial presentation for patients of the scheduled versus the on-demand cohort (undefined vs. 12 y; *P* = 0.005) (Figure 2D-E).

After the exclusion of all patients with a second ERCP within < 3 mo in both cohorts, patients in the scheduled cohort showed a tendency to a better TFS compared to patients in the on-demand cohort (median TFS 16 vs. 13 y; *P* = 0.083) (Supplemental Figure 2A, http://links.lww.com/HC9/B11). Further subgroup analysis, in which we excluded all patients with a second ERCP within < 3 mo showed a superior TFS for patients of the scheduled cohort compared to patients in the on-demand cohort since initial presentation (median TFS 16 vs. 13 y; *P* = 0.029) (Supplemental Figure 2B, http://links.lww.com/HC9/B11).

Mirroring the higher rate of LTs in the on-demand cohort, Kaplan-Meier curve analyses of overall survival did not show differences between both cohorts (since initial diagnosis: *P* = 0.848; since initial presentation: *P* = 0.657). Accordingly, no difference was observed in 5-, 10-, or 15-year overall survival rates (Supplemental Figure 3A,B, http://links.lww.com/HC9/B11). This is expected, given the high posttransplant survival in this indication.^32^

### Scheduled program as an independent predictor for TFS

Since both cohorts may be differently affected by confounders, we next aimed to analyze predictors associated with TFS. We performed univariate and multivariate analyses for inclusion in our scheduled program, gender, age at diagnosis, relevant stenosis at initial ERCP, presence of IBD, presence of overlap syndrome, and baseline ALP ≥ 2.5 ULN. Univariate and multivariate analysis confirmed that adherence to our scheduled program is an independent predictor for TFS (HR 0.580 [0.349–0.965]; *P* = 0.036). Further, a statistical significance in multivariate Cox regression analysis could be revealed for age at initial diagnosis (HR 1.028 [1.008–1.049]; *P*=0.007) and baseline ALP ≥ 2.5 ULN (HR 1.803 [1.077–3.019]; *P*=0.025) (Table 2).

**TABLE 2:** Univariate and multivariate Cox regression analysis for transplant-free survival since initial diagnosis in the scheduled versus on-demand cohort

	Univariate Cox regression		Multivariate Cox regression #1	
Parameter	*P*-value	HR (95% CI)	*P*-value	HR (95% CI)
Scheduled program	**0.025**	0.574 (0.354, 0.931)	**0.036**	**0.580 (0.349**, **0.965)**
Age at initial diagnosis	**0.001**	1.032 (1.012, 1.052)	**0.007**	1.028 (1.008, 1.049)
Gender (female)	0.851	1.052 (0.622, 1.779)	—	—
Overlap syndrome with AIH	0.818	0.924 (0.470, 1.815)	—	—
Inflammatory bowel disease	0.457	1.307 (0.646, 2.642)	—	—
Baseline ALP ≥2.5 ULN	**0.015**	1.894 (1.132, 3.169)	**0.025**	1.803 (1.077, 3.019)
Relevant Stenosis at initial ERCP	0.955	0.984 (0.565, 1.713)	—	—

*Note:* Significant results (*P* < 0.05) are shown in bold type.

Abbreviations: AIH, autoimmune hepatitis; ALP, alkaline phosphatase; ERCP, endoscopic retrograde cholangiopancreaticography; ULN, upper limit of normal.

### Development of hepatic decompensation, recurrent cholangitis episodes, and hepatobiliary malignancies

**FIGURE 3:**
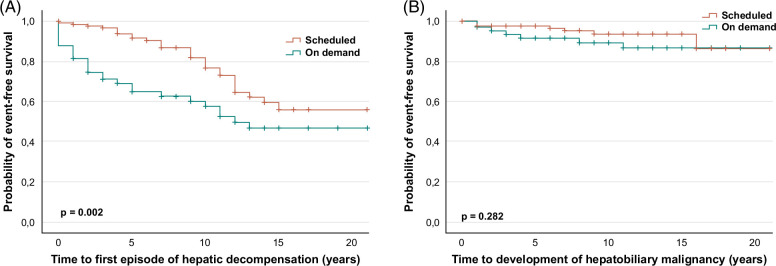
Time to development of the first episode of hepatic decompensation and time to development of hepatobiliary malignancy. Kaplan-Meier analysis reveals a significantly longer time to the first episode of hepatic decompensation in the scheduled cohort (red curve) compared to the on-demand cohort (*P* = 0.002) (A). No significant association could be observed when analyzing the time to development of hepatobiliary malignancies between both cohorts (*P* = 0.282) (B); (*P < 0.05; **P < 0.01; ***P < 0.001).

During the course of the disease, more patients developed episodes of hepatic decompensation in the on-demand cohort (21% vs. 44%, *P* < 0.001; Supplemental Table 2, http://links.lww.com/HC9/B11). Moreover, hepatic decompensation occurred earlier in those patients (*P* = 0.002) (Fig. 3A; Supplemental Table 3, http://links.lww.com/HC9/B11). Hepatic decompensation involved episodes of development of ascites, SBP, variceal bleeding, hepatic encephalopathy, and hepatorenal syndrome. Analysis of these particular factors individually revealed a more frequent occurrence of ascites (scheduled: 17% vs. on demand: 33%; *P* = 0.009), variceal bleeding episodes (scheduled: 6% vs. on demand: 17%; *P* = 0.018) and SBP (scheduled: 3% vs. on demand: 11%; *P* = 0.029) in the on-demand cohort. Moreover, hepatorenal syndrome revealed a tendency towards lower frequency of occurrence and time of the event in the scheduled cohort (Supplemental Table 2, http://links.lww.com/HC9/B11, and 3, Supplemental Figure 4, http://links.lww.com/HC9/B11). Notably, frequently recurring cholangitis episodes (≥ 2 and ≥ 3 episodes) with the necessity of hospitalization and i.v. antibiotic treatment occurred more often in the on-demand cohort (≥ 2 episodes: scheduled: 12% vs. on demand: 26%; *P* = 0.011; ≥ 3 episodes: scheduled: 5% vs. on demand: 18%; *P* = 0.003). Development of a hepatobiliary malignancy was similar in both cohorts (5% vs. 10%; *P* = 0.185) (Figure 3B; Supplemental Table 2, http://links.lww.com/HC9/B11).

### Procedure-associated adverse events

Analyzing 794 procedures, we documented 34 procedure-associated adverse events, corresponding to an overall complication rate of 4% in our total PSC population. The overall rate of adverse events was similar in both cohorts (scheduled: 4% vs. on demand 6%, not significant). The occurrence rate of post-ERCP–cholangitis, post-ERCP–pancreatitis, and post-ERCP–bleeding episodes was similar in both cohorts. Only bile duct perforation occurred more often in the on-demand cohort (scheduled: 0 vs. on-demand: 2/179; *P* = 0.043). Even though a trend for higher complication rates related to the first ERCP (vs. follow-up procedures) was observed, the impact was not statistically significant (first ERCP: 7% vs. following ERCPs 3%; *P* = 0.499). The majority of adverse events were classified as “mild.” Only 2 adverse events were classified as severe: 1 case of severe pancreatitis with the necessity of intensive care treatment and 1 case of bile duct perforation with the necessity of a re-intervention. No patient died due to adverse events associated with applied endoscopic procedures. Procedure-associated adverse events in the entire cohort and subgroups are presented in Supplemental Table 4, http://links.lww.com/HC9/B11.

### Progression between the first and second ERCP predicts survival outcome

**FIGURE 4:**
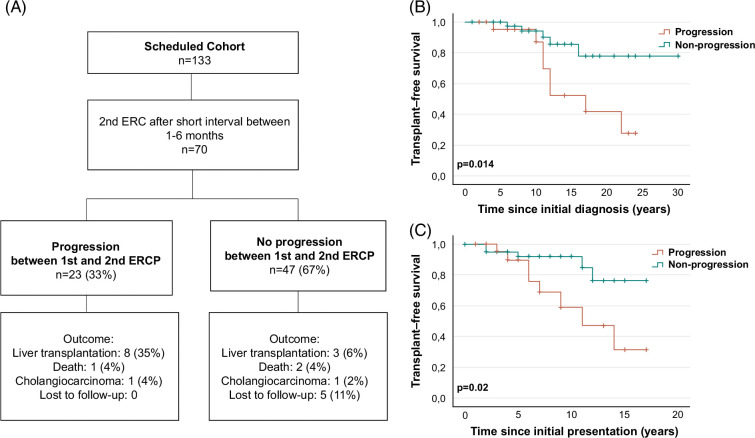
Outcome analysis and transplant-free survival in the progression versus nonprogression cohort. (A) Flowchart with an overview of outcome analysis results in the progression versus nonprogression cohort. Kaplan-Meier analysis of transplant-free survival (TFS) in the progression (orange curve) versus nonprogression (green curve) cohort revealed a poorer outcome for the progression cohort since initial diagnosis (median TFS: 17 y vs. undefined; *P* = 0.014) (B) and since initial presentation (median TFS: 11 y vs. undefined; *P* = 0.02) (C).

Despite the beneficial outcome in the scheduled ERCP cohort, some of these patients with PSC developed a progressive disease. We, therefore, hypothesized that the endoscopic findings of subsequent procedures would be predictive of the future disease course. Thus, we retrospectively analyzed all patients within our scheduled cohort who received a second ERCP within 6 months after the initial ERCP. Our findings revealed that 23 patients (33%) showed a progression in ERCP findings between the first and second ERCP (progression cohort). “Progression“ was defined as an increase in the number of stenoses, progression in the extent of stenosis, the increase in rarefication of the bile duct system, the necessity of a stent implantation, or the development of an acute non-ERCP–related cholangitis episode. Forty-seven patients (67%) revealed a response to treatment or stable findings (nonprogression cohort) (Figure 4A).

During the course of the disease, 35% of patients in the progression cohort received an LT, 4% died from a liver-related death, and 4% developed CCA. In contrast, in the nonprogression cohort, only 6% received an LT, 4% died from a liver-related death, and 2% developed CCA (Figure 4A). Kaplan-Meier survival curve analysis revealed a significantly impaired median TFS since initial presentation and initial diagnosis for patients of the progression cohort compared to patients in the nonprogression cohort (since initial diagnosis: 17 y vs. undefined; *P* = 0.014; since initial presentation: 11 y vs. undefined; *P* = 0.02) (Figure 4B,C). Additional analysis of TFS of the on-demand cohort versus progression cohort did not reveal a difference between both cohorts (Supplemental Figure 5, http://links.lww.com/HC9/B11).

### Baseline patient and disease characteristics of the progression versus nonprogression cohort

At the time of initial ERCP, no significant differences could be detected between the progression and nonprogression cohorts of the scheduled group with respect to median age at initial diagnosis, gender, medication, body mass index, and comorbidities such as arterial hypertension or diabetes. Only overlap syndrome occurred more frequently in the progression cohort but was overall very low (n = 4 (17%) vs. n = 1 (2%); *P* = 0.020). In contrast, the presence of IBD was equally distributed between both cohorts (n = 16 (70%) vs. n = 33 (70%); not significant). Baseline laboratory values did not show a significant difference between both cohorts; however, ALP, gamma-glutamyl transferase, alanine transaminase, and aspartate aminotransferase levels tended to be higher in the progression cohort. The median number of ERCPs per patient was higher in the progression cohort compared to the nonprogression cohort (median: 10 (4–20) vs. 7 (2–25); *P* = 0.015). With regard to initial ERCP findings, no difference could be found with respect to the time of initial diagnosis to ERCP, presence of stenosis at initial ERCP, stenosis morphology, and affection of the biliary tract system (Table 3). Further baseline characteristics are listed in Table 3.

**TABLE 3:** Baseline patient and disease characteristics: progression vs. nonprogression cohort

	Progression n = 23 (%)	Nonprogression n = 47 (%)	*P*-value
Gender	—	—	0.763
** **Male	16 (70)	31 (66)	—
** **Female	7 (30)	16 (34)	—
Median age at initial diagnosis (range)	31 (15–57)	32 (12–65)	0.945
Mean BMI (SD)	24.8 ± 3.6	24.6 ± 4.4	0.885
Secondary illness			
** **Arterial hypertension	8 (35)	10 (21)	0.225
** **Diabetes	2 (9)	1 (2)	0.203
Overlap syndrome with			
** **AIH	4 (17)	1 (2)	**0.020**
Presence of IBD	16 (70)	33 (70)	0.956
** **Ulcerative colitis	15 (65)	27 (57)	—
** **Crohn’s disease	0 (0)	5 (11)	—
** **Undetermined	1 (4)	1 (2)	—
UDCA^a^	23 (100)	47 (100)	—
Median number of performed ERCPs per patient (range)	10 (4, 20)	7 (2, 25)	**0.015**
Stenosis amenable for intervention at initial ERCP	17 (74)	40 (85)	0.258
Manifestation^a^:	—	—	0.311
** **Exclusively intrahepatic	2 (9)	10 (22)	—
** **Exclusively extrahepatic	3 (13)	3 (7)	—
** **Intrahepatic and extrahepatic	18 (78)	33 (72)	—
Affection of the biliary tract system^b^:	—	—	0.716
** **Right-sided	3 (15)	5 (12)	—
** **Left-sided	3 (15)	10 (24)	—
** **Both	14 (70)	27 (64)	—
Median baseline laboratory parameters (Min-Max)			
** **Bilirubin [mg/dL]	0.7 (0.5, 4.4)	0.8 (0.2, 4.7)	0.549
** **ALT [U/L]	83 (28, 232)	54 (14, 456)	0.401
** **AST [U/L]	66 (25, 115)	47 (18, 246)	0.468
** **ALP [U/L]	261 (70, 626)	184 (61, 798)	0.410
** **GGT [U/L]	302 (38, 956)	175 (17, 1276)	0.458
** **Creatinine [mg/dL]	0.8 (0.4, 1)	0.8 (0.5, 1.2)	0.696
** **INR	1 (0.9, 1.1)	1 (0.9, 1.2)	0.763

*Notes:* Data are n (%) of patients if not indicated otherwise. The percentages were rounded and may not sum 100%. Significant results (*P*<0.05) are shown in bold type.

Laboratory reference values: Reference values: Bilirubin < 1.2 mg/dL, ALT < 31 U/L, AST 35 U/L, ALP 35–105 U/L, GGT 5–36 U/L, creatinine 0.5–0.96 mg/dL, INR 0.9–1.25

^a^ Manifestation of stenosis was analyzed in 69 patients; 23 patients in the progression cohort and 46 patients in the nonprogression cohort.

^b^ Affection of the biliary tract system was analyzed in 62 patients; 20 patients in the progression cohort and 42 patients in the nonprogression cohort.

Abbreviations: AIH, autoimmune hepatitis; ALP, alkaline phosphatase; ALT, alanine transaminase; AST, aspartate aminotransferase; BMI, body mass index; ERCP, endoscopic retrograde cholangiopancreatography; GGT, gamma-glutamyl transferase; IBD, inflammatory bowel disease; INR, international normalized ratio; PBC, primary biliary cholangitis; UDCA, ursodeoxycholic acid.

The progression cohort not only had a poorer outcome with respect to TFS, but also a higher occurrence rate of cholangitis episodes (≥1 episode: progression: 48% vs. nonprogression: 11%; *P*=0.001; ≥2 episodes: progression 22% vs. nonprogression: 2%; *P*=0.007). However, presence of frequently recurrent (≥3) cholangitis episodes was equally distributed between both cohorts. Development of hepatic decompensation and time to decompensation did not differ significantly between both cohorts and only showed a tendency to more frequent events in the progression cohort. This finding might be explained by the small sample size.

To identify predictors that are associated with TFS, we performed a Cox regression analysis. Univariate and multivariate analysis confirmed that progression between first and second ERCP is an independent risk factor for TFS (HR 3.643 (1.151–11.526); *P* = 0.028). The presence of overlap syndrome with autoimmune hepatitis did not reveal a significant association with respect to TFS (HR 0.977 (0.206–4.626); *P* = 0.977)) in multivariate analysis. Of note, age at initial diagnosis, IBD, gender, baseline ALP ≥ 2.5 ULN and presence of overlap syndrome did not show a significant association in univariate analysis either (Supplemental Table 5, http://links.lww.com/HC9/B11). Consequently, our data reveals that only endoscopic progression between the first and second ERCP, but no other baseline factor identifies the adverse TFS.

## DISCUSSION

Despite the rather cautious use of endoscopic interventions with ERCP due to its invasiveness and reported risk for complications, several studies have established the beneficial effects of ERCP in patients with PSC.^8,11,23,33,34^ However, whether patients should receive their follow-up interventions only on demand or whether they benefit from regular endoscopic interventions is not clear. Our data reveal encouraging outcome data from a cohort that was enrolled in a scheduled endoscopic program. This approach indicated longer TFS and lower rates of hepatic decompensation and recurrent cholangitis episodes. These retrospective long-term data should be used as a basis for prospective multicenter studies evaluating scheduled interventional programs for patients with PSC. To our knowledge, there is only 1 comparable study from another high-volume endoscopic center where a similar approach to ours has been evaluated, revealing that stricture treatment is beneficial even in asymptomatic patients and can improve TFS.^12^ In their large (n = 286) retrospective study from Heidelberg, Rupp, and colleagues compared scheduled versus on-demand ERCPs based on patient preferences and demonstrated a superior TFS in patients with dominant stenosis receiving endoscopic interventions on a regular basis. In line with our results, they revealed lower death and transplantation rates in the scheduled cohort.^12^ Median 5-year and 10-year survival rates since initial presentation were comparable in both studies, although 2 different approaches with respect to the timing of initial ERCP and time intervals between interventions were used. While we performed initial ERCP according to guideline-based criteria, Rupp et al carried out endoscopic treatment in all patients at entry into the study independently of clinical and radiographic characteristics. Moreover, time intervals between endoscopic interventions were different: Rupp and colleagues repeated balloon dilatation after 4 weeks, 3 months, and 6 months up to the morphological resolution, and subsequently, patients underwent diagnostic ERCP at yearly intervals. In contrast, we performed follow-up ERCPs at regular intervals of 6 months with further adjustments based on clinical and radiographic data. Notably, the average number of performed ERCPs in both studies was similar. Both studies with 2 different approaches indicate that scheduled ERCP programs in experienced high-volume endoscopy centers are beneficial. Our data were obtained retrospectively. In light of the similar data from Rupp et al, they should be used as a basis for further prospective randomized studies to explore the ideal time point for initial ERCP as well as the intervals of follow-up interventions.

One potential argument for a restrictive use of ERCPs is the risk of endoscopy-induced cholangitis episodes, which is derived from the idea that ERCP may introduce bacteria in partially obstructed but previously noninfected intrahepatic bile ducts, creating a situation of increased risk with limited benefit.^35^ Our results reveal a relatively low risk of recurrent cholangitis episodes in patients who underwent a scheduled ERCP. Thus, regular ERCP may indeed improve the biliary flow and prevent microbial outgrowth in the biliary tract. Our overall complication rate of 4% is within the range of previous studies, which report overall complication rates between 2% and 6%.^11,33,34,36,37^ More importantly, the majority of our procedure-associated adverse events could be classified as “mild.” Thus, since an increased number of endoscopic interventions within a scheduled program is not associated with an increased complication rate, a higher complication rate should not be considered as a reason for the restrictive use of endoscopic interventions. Another rising concern about repeated endoscopy is the high number of recent outbreaks of duodenoscope-associated multidrug-resistant organisms, which has drawn attention to the risk of transmission and spread of multidrug-resistant organisms during repeated duodenoscope procedures. Recent studies have proven that strict accordance with manufacturer and reprocessing guidelines does not serve a guarantee to reliably eliminate soil or bioburden, allowing transmission of multidrug-resistant organisms.^38^ Nevertheless, evaluation and report of multidrug-resistant organisms during endoscopic procedures should be part of future prospective randomized studies for evaluation of follow-up ERCP programs.

We report that the comparative findings from the first 2 ERCPs performed within an interval of 6 months can reveal t2 different PSC “activity” types with a remarkably different outcome. Our data demonstrate that patients with a progression between the first 2 ERCPs had a significantly worse TFS compared to patients without progression in ERCP findings. It should be noted that this subgroup of high-risk patients received an intensive endoscopic follow-up, and the TFS of patients in this group was still not inferior to the entire on-demand group. This fact underlines the importance of performing scheduled endoscopic interventions and may indicate that even in a cohort with a predicted poor outcome, better survival rates may be achieved.

We did not detect significant differences between both the progression and nonprogression cohorts with respect to baseline clinical characteristics. Only the presence of overlap syndrome occurred more frequently in the progression cohort; however, the total number of patients affected was very low. In addition, univariate Cox regression analysis did not reveal the presence of an overlap syndrome to be an independent risk factor for TFS. Contrary to our findings, there are previous studies that indicate that the clinical course of patients with PSC/autoimmune hepatitis overlap syndrome appears to be beneficial compared to classical PSC, which was attributed to the positive influence of immunosuppression on the progression of the PSC component.^39–41^ However, a large retrospective study of the International PSC study group found no significant difference in TFS between the PSC/AIH variant versus the classical PSC sub-phenotype.^26^

Regardless of the overlap syndrome, further experimental studies should explore the biological basis for the different responses to ERCP treatment. Possible explanations might be an underlying different immune environment or also the presence of 2 disease activity types concordant to IBD (inactive vs. active). Moreover, recent findings indicate that PSC is characterized by an altered microbiome.^42^ In this context, recent studies revealed significantly poorer outcomes in patients with a high abundance of *Enterococcus* species (spp.). and *Candida* spp. in bile culture samples.^43,44^ Thus, it appears reasonable to conduct further mechanistic studies investigating the role of the microbiota for disease activity and responsiveness to endoscopic treatment.

The main limitations of our study are the retrospective design and the lack of randomization regarding endoscopic treatment. To overcome potential selection bias, we performed propensity score matching and subsequent Cox regression analysis, which confirmed our findings. However, due to the retrospective design, potential bias cannot be entirely excluded and therefore the definitive conclusions require prospective trials. Since our on-demand cohort included all patients who were not able to be part of our scheduled program (eg, due to geographical reasons), it is possible that in addition to the differences in ERCP approaches between these groups, other confounders might have affected the outcome. Specifically, surveillance parameters and frequency of clinical laboratory and imaging studies should be standardized, and patients should be attributed to both cohorts under controlled-randomized conditions to avoid selection bias. Although not statistically significant, the on-demand cohort included a higher number of patients with intrahepatic strictures who are less likely to benefit from ERCP. Further, a slight tendency of higher MELD score values was present in the on-demand cohort, which we considered in our propensity score matching to overcome this potential bias. Follow-up duration and loss to follow-up numbers in both cohorts were similar. Another potential limitation is the mono-center trial design, which may limit the translatability of our findings to centers with a lower frequency of interventions and less experienced endoscopists. Nonetheless, the single-center design ensured a standardized endoscopic approach with well-defined interventions and adherence to our in-house algorithm for the management of patients with PSC.

In conclusion, we report here the outcome data of a scheduled follow-up ERCP program cohort for patients with PSC in an experienced high-volume endoscopy center. Our data suggest the initiation of multicenter randomized controlled prospective trials to explore the full potential of regular endoscopic treatment as a strategy to prevent disease progression in patients PSC.

## Supplementary Material

## Data Availability

Data are available upon request from the Department of Hepatology and Gastroenterology of the Charité University Medicine Berlin for researchers who meet the criteria for access to confidential data (gastro-cvk@charite.de). The informed consent of patients was waived due to the retrospective nature of the study.
