# Supplementary material for: Long-term impact of scheduled regular endoscopic interventions for patients with primary sclerosing cholangitis
Source: Hepatol Commun. 2024 Sep 3;8(9):e0494. doi: 10.1097/HC9.0000000000000494 (PMC11371311; doi:10.1097/HC9.0000000000000494)
Supplement: Supplementary file 1 [file hc9-8-e0494-s001.pdf]

Supplementary Data

Supplementary Figure 1. Outcome analysis and transplant-free survival in the scheduled vs. on demand cohort. Kaplan-Meier analysis of transplant-free survival (TFS) in the scheduled (red curve) vs. on demand cohort (blue curve) since first Endoscopic retrograde cholangiopancreatography (ERCP) revealed a beneficial outcome for the scheduled cohort (median TFS: 16 years vs. 9 years; $p<0.001$); (* $p < 0.05$; ** $p < 0.01$; *** $p < 0.001$).

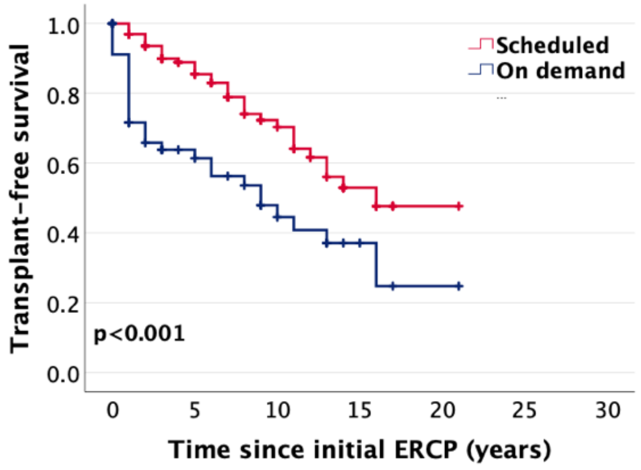

Supplementary Fig. 2: A. After exclusion of all patients with a 2nd ERCP within <3 months in both cohorts, patients in the scheduled cohort (n=89) showed a tendancy to a better transplant-free survival compared to patients in the on demand cohort (n=46) since initial presentation (median TFS 16 vs. 13 years; p=0.084). Further subgroup analysis, in which we excluded all patients with a 2nd ERCP within <3 months showed a superior TFS for patients of the scheduled cohort compared to patients in the on demand cohort since initial presentation (median TFS 16 vs. 13 years; p=0.029); (* p < 0.05; ** p < 0.01; *** p < 0.001).

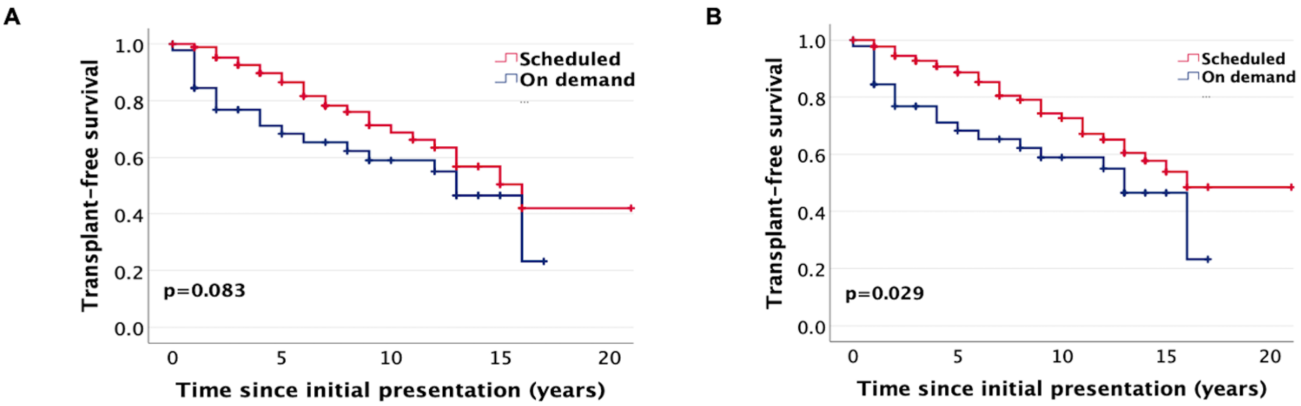

Supplementary Figure 3: Overall survival since initial diagnosis and initial presentation scheduled vs. on demand cohort. Kaplan-Meier analysis of overall survival since initial diagnosis between the scheduled cohort (red curve) and the on demand cohort (blue curve) revealed no significant difference (median survival undefined; p=0.848). The 15-year survival rate was 90% vs. 87% (5-year survival rate 99% vs. 99%; 10-year survival rate: 95% vs. 89%; 20-year survival rate: 80% vs. 81%) (**A**). Overall survival since initial presentation did not show a significant difference between both cohorts (median survival undefined; p=0.657). The 5-year survival rate was 95% vs. 91% (10-year survival rate: 89% vs. 87%, 15-year survival rate: 72% vs. 74%; 20-year survival rate: 72% vs. 63%) (**B**); (* $p < 0.05$; ** $p < 0.01$; *** $p < 0.001$).

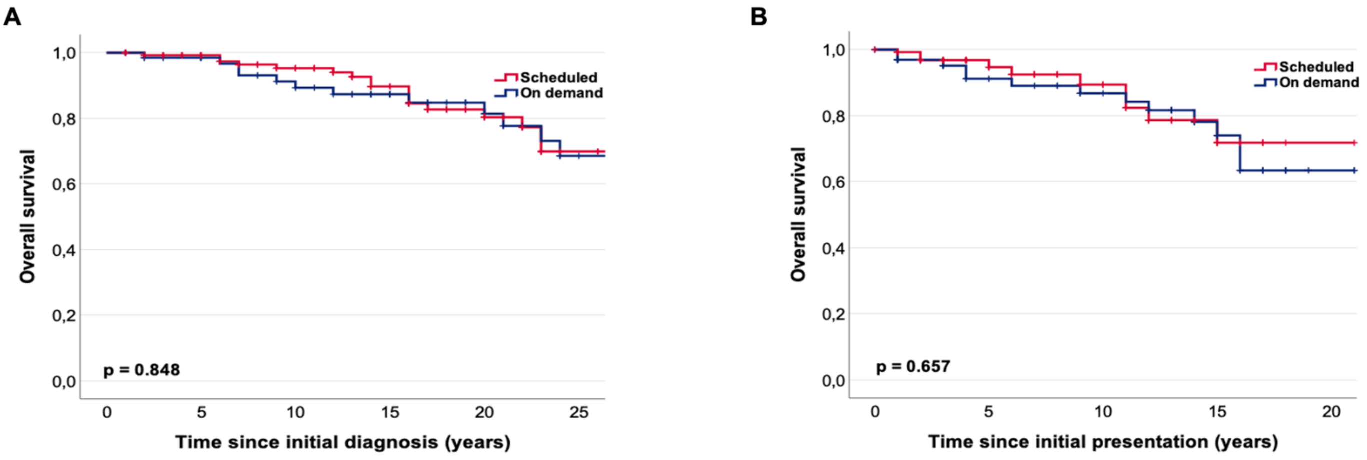

Supplementary Figure 4: Time to development of first episode of hepatic decompensation. Kaplan-Meier analysis revealed a significantly longer time to first episode of development of ascites after initial presentation in the scheduled cohort (red curve) compared to the on demand cohort (p=0.028) (**A**). No significant correlation could be observed when analyzing time to development of spontaneous bacterial peritonitis (p=0.063) (**C**), variceal bleeding (p=0.129) (**D**), hepatic encephalopathy (p=0.785) (**E**) and hepatorenal syndrome (p=0.062) (**F)**; (* $p < 0.05$; ** $p < 0.01$; *** $p < 0.001$).

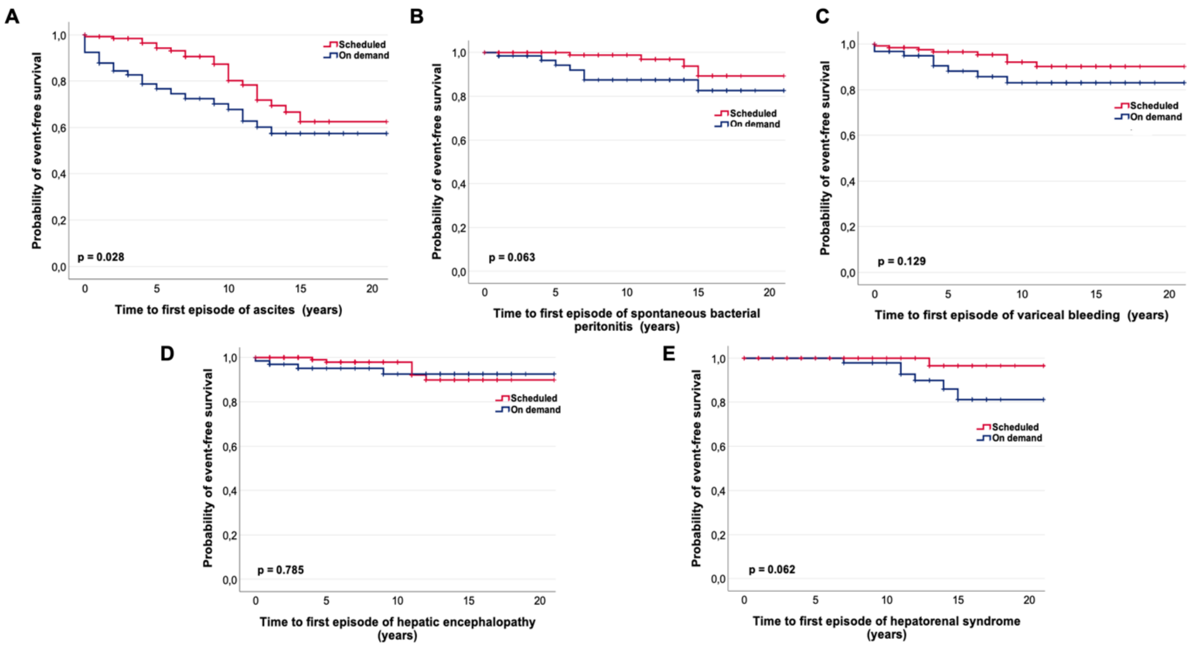

Supplementary Figure 5: Transplant-free survival since initial diagnosis (A) and initial presentation (B) in the on demand vs. progression cohort. Median transplant-free survival (TFS) since initial diagnosis did not reveal a significant difference between the on demand cohort (blue curve) and progression cohort (orange curve) (median TFS: on demand: 19 years vs. progression: 17 years; p=0.703). The 15-year survival rate since initial diagnosis was 61% vs. 52% (5-year survival rate 95% vs. 95%; 10-year survival rate: 78% vs. 87%; 20-year survival rate: 50% vs. 42%) (**A**). In line, no significant difference in TFS since initial presentation could be revealed between both cohorts (median TFS: on demand: 12 years vs. progression:

11 years; p=0.384). 5-year survival rate: 67% vs. 90%; 10-year survival rate: 54% vs. 59%, 15-year survival rate: 42% vs. 32% (**B**).

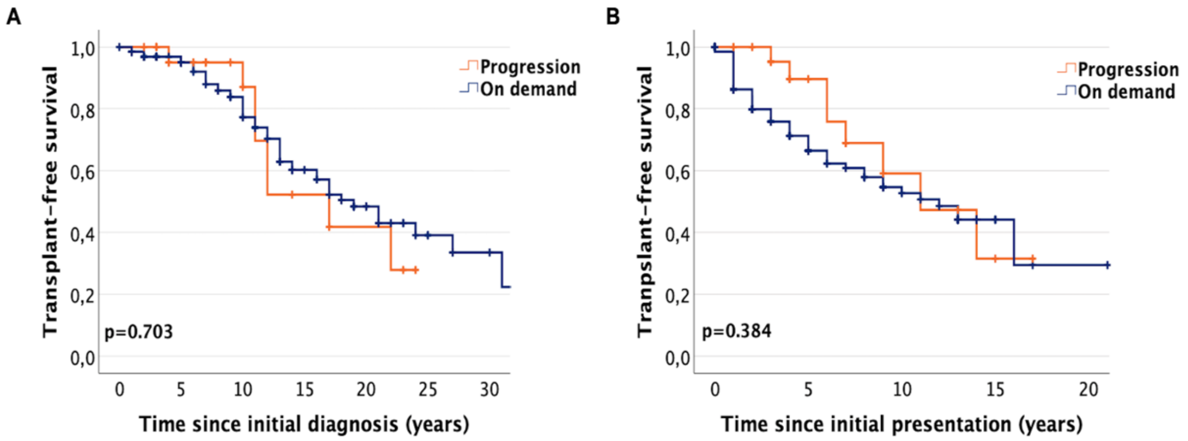

Supplementary Tables

Supplementary Table 1: Definition and classification into grade of procedure-associated adverse events

**Degree of severity**	**Post-ERCP pancreatitis:**<br>New onset of abdominal pain and a ≥ 3-fold elevation of serum lipase levels within two weeks after the procedure	**Post-ERCP cholangitis:**<br>Fever, leukocytosis and / or positive blood culture necessitating the use of antibiotics	**Post-ERCP bleeding:**<br>Hemorrhage leading to blood transfusion or re-intervention	**Post-ERCP perforation:**<br>Extravasation of contrast material on radioscopy requiring re-intervention and / or stenting
**Mild**	▪ No organ failure<br>▪ No local or systemic complications	No criteria of moderate/severe cholangitis.	▪ Abortion of the procedure<br>▪ Unplanned admission < 4 nights	▪ Abortion of the procedure<br>▪ Unplanned admission < 4 nights
**Moderate**	▪ Organ failure <48 h and/or<br>▪ Local or systemic complications without persistent organ failure	Any of the following:<br>▪ white blood cell count > 12 000 or < 4000/mm^3,<br>▪ fever ≥ 39 °C,<br>▪ age ≥ 75 years,<br>▪ total bilirubin ≥ 5 mg/dl<br>▪ hypoalbuminemia	Any of the following:<br>▪ Unplanned admission 4-10 nights<br>▪ ICU admission 1 night<br>▪ Need for transfusion<br>▪ Repeat endoscopy or interventional radiology<br>▪ Intervention for integument injuries	Any of the following:<br>▪ Unplanned admission 4-10 nights<br>▪ ICU admission 1 night<br>▪ Need for transfusion<br>▪ Repeat endoscopy or interventional radiology<br>▪ Intervention for integument injuries
**Severe**	Persistent (48 hours) organ failure	Dysfunction of at least one of the following systems:<br>▪ cardiovascular<br>▪ neurological	Any one of the following:	Any one of the following:

	▪ respiratory<br>▪ renal<br>▪ hepatic<br>▪ hematological system	▪ Unplanned admission >10 nights<br>▪ ICU admission >1 night<br>▪ Need for surgery<br>▪ Permanent disability	▪ Unplanned admission >10 nights<br>▪ ICU admission >1 night<br>▪ Need for surgery<br>▪ Permanent disability

Definition and classification into grade of procedure-associated adverse events according to recommendations of the 2018 revised Tokyo Guidelines and the lexicon of definitions proposed in 2010 for the American Society for Gastrointestinal Endoscopy [13, 15].

Supplementary Table 2: Hepatic decompensation, recurrent cholangitis episodes and development of hepatobiliary malignancies in the scheduled vs. on demand cohort

	**All patients**<br>n=201	**Scheduled cohort**<br>n=133	**On demand cohort**<br>n=68	**P scheduled vs. on demand**
**Hepatic decompensation***	57 (29%)	28 (21%)	29 (44%)	**<0.001**
**Ascites***	44 (22%)	22 (17%)	22 (33%)	**0.009**
**Variceal bleeding***	19 (10%)	8 (6%)	11 (17%)	**0.018**
**Spontaneous bacterial peritonitis***	11 (6%)	4 (3%)	7 (11%)	**0.029**
**Hepatic encephalopathy***	10 (5%)	6 (5%)	4 (6%)	0.655
**Hepatorenal syndrome***	10 (5%)	4 (3%)	6 (9%)	0.068
**Presence of ≥ 1 acute cholangitis episode****	57 (30%)	34 (26%)	23 (37%)	0.113
**≥ 2 cholangitis episodes****	31 (16%)	15 (12%)	16 (26%)	**0.011**
**≥ 3 cholangitis episodes****	17 (9%)	6 (5%)	11 (18%)	**0.003**
**Hepatobiliary malignancy**	14 (7%)	7 (5%)	7 (10%)	0.185

Data are n (%) of patients, if not indicated otherwise. The percentages were rounded and may not sum 100%. Significant results ($p<0.05$) are shown in bold type. *Hepatic decompensation (ascites, variceal bleeding, spontaneous bacterial peritonitis, hepatic encephalopathy, hepatorenal syndrome) was analyzed in 197 patients; 131 patients in the scheduled cohort and 66 patients in the on demand cohort. **Number of cholangitis episodes was analyzed in 193 patients; 131 patients in the scheduled cohort and 62 patients in the on demand cohort.

Supplementary Table 3: Time to first hepatic decompensation and to development of hepatobiliary malignancies. Number of patients that developed hepatic decompensation, ascites, variceal bleeding, hepatic encephalopathy, hepatorenal syndrome and hepatobiliary malignancies after 3, 5, 10, 15 and 20 years in the scheduled vs. on demand cohort.

**Time to first decompensation since initial presentation**	**Scheduled cohort**<br>n=133	**On demand cohort**<br>n=68	**p-value log-rank**
**Hepatic Decompensation***			**0.002**
▪ After 3 years	4 (3%)	18 (27%)	
▪ After 5 years	9 (7%)	21 (32%)	
▪ After 10 years	19 (16%)	24 (36%)	
▪ After 15 years	28 (21%)	28 (42%)	
▪ After 20 years	28 (21%)	28 (42%)	

**Ascites***			**0.028**
▪ After 3 years	2 (2%)	11 (17%)	
▪ After 5 years	6 (5%)	14 (21%)	
▪ After 10 years	15 (11%)	18 (27%)	
▪ After 15 years	22 (17%)	22 (33%)	
▪ After 20 years	22 (17%)	22 (33%)	
**Spontaneous bacterial peritonitis***			0.063
▪ After 3 years			
▪ After 5 years	0 (0%)	1 (2%)	
▪ After 10 years	0 (0%)	3 (5%)	
▪ After 15 years	1 (1%)	6 (9%)	
▪ After 20 years	4 (3%)	7 (11%)	
	4 (3%)	7 (11%)	
**Variceal bleeding***			0.129
▪ After 3 years	3 (2%)	3 (5%)	
▪ After 5 years	4 (3%)	6 (9%)	
▪ After 10 years	7 (5%)	8 (12%)	
▪ After 15 years	8 (6%)	8 (12%)	
▪ After 20 years	8 (6%)	8 (12%)	
**Hepatic encephalopathy***			0.785
▪ After 3 years	0 (0%)	3 (5%)	
▪ After 5 years	2 (2%)	3 (5%)	
▪ After 10 years	2 (2%)	4 (6%)	
▪ After 15 years	6 (5%)	4 (6%)	
▪ After 20 years	6 (5%)	4 (6%)	
**Hepatorenal syndrome***			0.062
▪ After 3 years	0	0	
▪ After 5 years	0	0	
▪ After 10 years	0	1 (2%)	
▪ After 15 years	1 (%)	6 (9%)	
▪ After 20 years	1 (%)	6 (9%)	
**Hepatobiliary malignancy**			0.282
▪ After 3 years	3 (2%)	4 (6%)	
▪ After 5 years	3 (2%)	5 (7%)	
▪ After 10 years	6 (4%)	6 (9%)	
▪ After 15 years	6 (4%)	7 (10%)	
▪ After 20 years	7 (5%)	7 (10%)	

Data are n (%) of patients, if not indicated otherwise. The percentages were rounded and may not sum 100%. Significant results ($p<0.05$) are shown in bold type. *Hepatic decompensation (ascites, spontaneous bacterial peritonitis, variceal bleeding, hepatic encephalopathy, hepatorenal syndrome) was analyzed in 197 patients; 131 patients in the scheduled cohort and 66 patients in the on demand cohort.

Supplementary Table 4: Secondary outcome analysis: post-ERCP adverse events

	**Entire cohort**	**Scheduled cohort**	**On demand cohort**	**p-value**
**No. of ERCPs analyzed**	794	615	179	
**No. Post-ERCP** adverse events				
**Overall**	34/794 (4%)	24/615 (4%)	10/179 (6%)	0.890
**Initial ERCP**	14/201 (7%)	8/133 (6%)	6/68 (9%)	
**Following ERCP**	20/593 (3%)	16/482 (3%)	4/111 (4%)	
**Post-ERCP cholangitis**				
**Overall**	6/794 (0.8%)	5/615 (1%)	1/179 (0.6%)	0.379
▪ Mild	▪ 6 (100%)	▪ 5 (100%)	▪ 1 (100%)	
▪ Moderate	▪ 0 (0%)	▪ 0 (0%)	▪ 0 (0%)	
▪ Severe	▪ 0 (0%)	▪ 0 (0%)	▪ 0 (0%)	
**Initial ERCP**	0 (0%)	0 (0%)	0 (0%)	
**Following ERCP**	6/593 (1%)	5/482 (1%)	1/111 (0,9%)	

**Post-ERCP pancreatitis**				
**Overall**	24/794 (3%)	18/615 (3%)	6/179 (3%)	0.356
▪ Mild	▪ 18 (75%)	▪ 15 (83%)	▪ 3 (50%)	
▪ Moderate	▪ 5 (21%)	▪ 3 (17%)	▪ 2 (33%)	
▪ Severe	▪ 1 (4%)	▪ 0 (0%)	▪ 1 (17%)	
**Initial ERCP**	11/201 (6%)	7/133 (17%)	4/68 (6%)	
**Following ERCP**	13/593 (2%)	11/482 (2.3%)	2/111 (2%)	
**Post-ERCP bleeding**				
Overall	2/794 (0.3%)	1/615 (0.2%)	1/179 (0.6%)	0.615
▪ Mild	▪ 1 (50%)	▪ 0 (0%)	▪ 1 (100%)	
▪ Moderate	▪ 1 (50%)	▪ 1 (100%)	▪ 0 (0%)	
▪ Severe	▪ 0 (0%)	▪ 0 (0%)	▪ 0 (0%)	
**Initial ERCP**	2/201 (1%)	1/133 (0.8%)	1/68 (2%)	
**Following ERCP**	0 (0%)	0 (0%)	0 (0%)	
**Bile duct perforation during ERCP**				
**Overall**	2/794 (0.3%)	0 (0%)	2/179 (1%)	**0.043**
▪ Mild	▪ 0 (0%)	▪ 0 (0%)	▪ 0 (0%)	
▪ Moderate	▪ 1 (50%)	▪ 0 (0%)	▪ 1 (50%)	
▪ Severe	▪ 1 (50%)	▪ 0 (0%)	▪ 1 (50%)	
**Initial ERCP**	1/201 (0.5%)	0 (0%)	1/68 (1%)	
**Following ERCP**	1/593 (0.2%)	0 (0%)	1/111 (1%)	

Data are n (%) of patients, if not indicated otherwise. The percentages were rounded and may not sum 100%. Endoscopic retrograde cholangiopancreaticography (ERCP)

Supplementary Table 5: Uni- and multivariate analysis of transplant-free survival of progression vs. non-progression cohort

	**Univariate Cox-Regression**		**Multivariate Cox-Regression**	
**Parameter**	**p-value**	**Hazard-Ratio (95% CI)**	**p-value**	**Hazard-Ratio (95% CI)**
**Progression between 1st and 2nd ERCP**	**0.024**	3.627 (1.186-11,098)	**0.028**	3.643 (1.151-11.526)
**Age at initial diagnosis**	0.314	1.022 (0.980-1.067)		
**Gender (female)**	0.395	0.570 (0.156-2.079)		
**Overlap Syndrome with AIH**	0.529	1.624 (0.359-7.348)	0.977	0.977 (0.206-4.626)
**Inflammatory Bowel Disease**	0.976	0.980 (0.269-3.567)		
**Baseline ALP ≥ 2.5 ULN**	0.051	3.334 (0.997-11.151)		

Bold values indicate significant values (p<0.05). Autoimmune Hepatitis (AIH); Alkaline phosphatase (ALP). Upper limit of Normal (ULN)
